# Robotic, laparoscopic, and open surgery for perihilar cholangiocarcinoma: a systematic review and direct pairwise meta-analysis

**DOI:** 10.3389/fsurg.2026.1825289

**Published:** 2026-09-18

**Authors:** Rui Cheng, Ling Su, Yudi Zhang, Jihui Zou, Zhengchun Zhu, Le Li, Zhonghua Liu

**Affiliations:** 1 Affiliated Chifeng Clinical College of Inner, Mongolia Medical University, Chifeng, China; 2 Department of Hepatobiliary Surgery, Chifeng Municipal Hospital, Chifeng, Inner Mongolia, China

**Keywords:** laparoscopic surgery, meta-analysis, open surgery, pairwise meta-analysis, perihilar cholangiocarcinoma, robot-assisted surgery, systematic review

## Abstract

**Background:**

Radical resection remains the cornerstone of curative-intent treatment for perihilar cholangiocarcinoma (pCCA). With the evolution of minimally invasive approaches, laparoscopic surgery (LS) and robot-assisted surgery (RAS) have been increasingly adopted in its surgical management. However, high-quality comparative evidence evaluating these approaches against conventional open surgery (OS) remains limited, and direct comparisons between LS and RAS are scarce. This systematic review and meta-analysis aimed to synthesize the available direct comparative evidence on RAS, LS, and OS for pCCA. Given the sparse evidence network and limited ability to verify the transitivity assumption, network meta-analysis was retained only as an exploratory adjunct.

**Methods:**

A systematic search of PubMed, Embase, the Cochrane Library, Web of Science, the China National Knowledge Infrastructure (CNKI), and the Wanfang Database was performed to identify clinical studies comparing robot-assisted surgery (RAS), laparoscopic surgery (LS), and open surgery (OS). Perioperative efficacy and safety were prespecified as the primary domains of interest. Outcomes included operative time, intraoperative blood loss, intraoperative transfusion rate, length of hospital stay, hospitalization costs, Clavien–Dindo grade ≥ III complications, R0 resection rate, 1- and 2-year overall survival, and changes in liver function and nutrition-related biochemical indices, including ALT, T-BIL, and ALB. The primary quantitative synthesis was based on direct pairwise meta-analyses of head-to-head comparisons, using random-effects or fixed-effect models according to prespecified heterogeneity criteria. An exploratory network meta-analysis was performed to describe the broader comparative evidence structure, but indirect comparisons and treatment rankings were not used as the primary basis for clinical interpretation. The certainty of evidence was evaluated using the GRADE framework, with additional consideration of network-specific concerns where applicable.

**Results:**

A total of 24 comparative studies involving 1,598 patients were included, comprising 18 LS–OS studies, five RAS–OS studies, and one direct RAS–LS study. Compared with OS, both LS and RAS were associated with significantly longer operative time, lower intraoperative blood loss, and shorter hospital stay. No clear differences were observed in intraoperative transfusion rate, R0 resection rate, 1-year overall survival, or 2-year overall survival. Neither LS nor RAS was associated with a statistically significant difference in major complications compared with OS. For RAS versus OS, the point estimate was greater than 1, but the confidence interval crossed the null value and the evidence was of very low certainty. For biochemical outcomes, LS was associated with a smaller postoperative–preoperative change in T-BIL, whereas no clear differences were observed in ALT or ALB. The single direct RAS–LS study was summarized using study-specific estimates and was not quantitatively pooled. The exploratory network analysis was generally directionally consistent with the direct pairwise evidence for several perioperative outcomes, but most outcome networks lacked a robust closed loop, and any triangular loop depended on a single small retrospective RAS–LS study. Key clinical effect modifiers, including tumor extent, Bismuth classification, vascular involvement, extent of hepatectomy, reconstruction requirements, case complexity, and surgeon or institutional experience, were incompletely reported across studies, limiting the validity of indirect comparisons. Reporting of operative extent, patient-selection criteria, surgeon experience, learning-curve status, and institutional surgical volume was incomplete and inconsistent, precluding reliable subgroup analysis or adjustment for these clinically important effect modifiers.

**Conclusion:**

Current direct comparative evidence suggests that minimally invasive surgery may offer selected short-term perioperative benefits in carefully selected patients with pCCA, particularly reduced intraoperative blood loss and shorter hospital stay with LS. However, the optimal selection criteria for minimally invasive approaches remain undefined. However, these potential benefits should be balanced against longer operative time and the low or very low certainty of the available evidence. No surgical approach can currently be considered superior with respect to intraoperative transfusion rate, major complications, oncologic outcomes, or survival. Evidence regarding liver function and nutrition-related biochemical indices remains limited and heterogeneous. Because the evidence network was sparse and the direct RAS–LS comparison was limited to a single small retrospective study, the transitivity assumption could not be adequately supported. Accordingly, network estimates and treatment rankings should be considered exploratory and hypothesis-generating rather than confirmatory and should not be used in isolation to guide surgical decision-making. Well-designed multicenter prospective studies with standardized reporting of tumor complexity, operative extent, reconstruction requirements, and surgeon or center experience are needed.

**Systematic Review Registration:**

https://www.crd.york.ac.uk/PROSPERO/view/CRD420261297194, PROSPERO: CRD420261297194.

## 1 Background

Perihilar cholangiocarcinoma (pCCA) represents the most common subtype of biliary tract malignancy, accounting for approximately 60%–67% of all cholangiocarcinomas. Originating at the confluence of the right and left hepatic ducts, it is a biologically aggressive tumor associated with a poor prognosis (1). Given its insidious onset and rapid progression, patients frequently present with obstructive jaundice or cholangitis, and a considerable proportion are diagnosed at a locally advanced stage or with vascular involvement, contributing to unfavorable overall outcomes. Complete surgical resection remains the only potentially curative treatment and the sole opportunity for long-term survival in pCCA (2, 3). Achieving an R0 resection typically necessitates hepatectomy with *en bloc* resection of the extrahepatic bile duct, regional lymphadenectomy, and biliary reconstruction (4, 5). When a margin-negative resection is achieved, survival improves substantially, with reported median overall survival ranging from 30 to 46 months and 5-year survival rates of 25%–40%, approaching 50% in selected series (6).

Open surgery has long been considered the standard approach for pCCA. These procedures often involve bile duct resection combined with major or extended hepatectomy, caudate lobectomy, and systematic lymphadenectomy, with vascular resection and reconstruction performed as indicated. Such operations are technically complex and highly invasive, with substantial risks of blood loss and transfusion, prolonged recovery, and serious postoperative complications—including bile leakage, liver failure, and infection—that can negatively impact postoperative quality of life and delay the initiation of adjuvant therapy (7). With the rapid advancement of minimally invasive surgery, both laparoscopic and robot-assisted techniques have increasingly been applied to complex hepatobiliary procedures, including pCCA. In theory, minimally invasive approaches provide magnified visualization and more precise dissection, which may reduce tissue trauma and intraoperative blood loss, mitigate postoperative pain and physiological stress, and promote earlier mobilization and recovery. Robotic systems may further overcome certain technical limitations of conventional laparoscopy by offering enhanced instrument articulation and stable three-dimensional visualization, potentially improving precision in confined operative fields—particularly during biliary-enteric anastomosis and vascular reconstruction (8, 9).

In this context, direct pairwise meta-analysis provides the most clinically interpretable synthesis of available head-to-head evidence. However, because the comparative evidence across RAS, LS, and OS is sparse and unevenly distributed, an exploratory network meta-analysis may provide additional descriptive information on the overall evidence structure. Importantly, such network-derived estimates rely on the transitivity assumption, which may be difficult to verify in the present setting because key clinical effect modifiers are incompletely reported. Therefore, network estimates and treatment rankings should be interpreted only as exploratory and hypothesis-generating rather than confirmatory. Accordingly, this study aimed to systematically review the available comparative evidence on RAS, LS, and OS for pCCA, with direct pairwise meta-analyses as the primary quantitative synthesis. In addition, given that patient selection represents a major determinant of outcomes in minimally invasive pCCA surgery, we sought to evaluate whether currently available comparative studies provided explicit and consistent criteria regarding indications, contraindications, and selection strategies for minimally invasive approaches. An exploratory network meta-analysis was additionally performed to describe the broader comparative evidence structure, but indirect comparisons and ranking results were not used as the primary basis for clinical interpretation. In addition, we sought to determine whether the available evidence provided sufficiently explicit and consistent criteria for selecting patients for a minimally invasive approach. Outcomes of interest included perioperative outcomes, postoperative safety, healthcare resource utilization, oncologic outcomes, liver function and nutrition-related biochemical indices, and short-term survival outcomes in patients undergoing radical resection for pCCA.

## 2 Materials and methods

This systematic review and meta-analysis was conducted in accordance with the Preferred Reporting Items for Systematic Reviews and Meta-Analyses 2020 statement. Because an exploratory network meta-analysis was additionally performed, relevant items from the PRISMA extension statement for network meta-analyses were also considered where applicable (Supplementary Table S1) (10). To enhance methodological transparency and reproducibility, the study protocol was prospectively registered in the International Prospective Register of Systematic Reviews (PROSPERO) (CRD420261297194). In the revised manuscript, direct pairwise meta-analyses were emphasized as the primary quantitative synthesis, whereas the network meta-analysis was retained only as an exploratory adjunct because the evidence network was sparse and the transitivity assumption could not be adequately verified.

### 2.1 Data sources and search strategy

A systematic literature search was conducted in PubMed, Embase, the Cochrane Library, Web of Science, China National Knowledge Infrastructure (CNKI), and the Wanfang Database from database inception to July 1, 2025. The search strategy combined controlled vocabulary and free-text terms related to perihilar cholangiocarcinoma and the relevant surgical approaches, including “perihilar cholangiocarcinoma”, “hilar cholangiocarcinoma”, “Klatskin tumor”, “laparoscopy”, “laparoscopic surgical procedures”, “robotic surgical procedures” and “robot-assisted surgery” as well as their Chinese equivalents (see Supplementary Table S2). No language restrictions were applied. Reference lists of included studies and relevant reviews were also screened manually, and citation tracking was performed to identify additional eligible studies. An updated search was performed before final study inclusion. Where outcome data were missing or insufficiently reported, attempts were made to contact the corresponding authors. Full database-specific search strategies and retrieval details are provided in the Supplementary Material. Because anatomical terminology is not fully uniform across the literature, study eligibility was based on a prespecified operational definition of pCCA, as detailed in the Selection Criteria.

### 2.2 Selection criteria

#### 2.2.1 Inclusion criteria

1. Population: Adults (≥18 years) with a confirmed diagnosis of perihilar cholangiocarcinoma undergoing intended curative resection. For study eligibility, perihilar cholangiocarcinoma was operationally defined as a malignancy arising at or centered on the biliary confluence of the right and left hepatic ducts, including lesions extending distally to the common hepatic duct and/or proximally to the second-order intrahepatic ducts. Studies that used the terms “hilar cholangiocarcinoma” or “Klatskin tumor” were included only if the reported tumor location, surgical description, or Bismuth classification was consistent with this perihilar definition. Mixed biliary tract cohorts were eligible only when pCCA-specific data could be extracted separately; otherwise, they were excluded.
2. Interventions and comparators: Studies directly comparing at least two of the following surgical approaches were eligible: robot-assisted surgery (RAS), laparoscopic surgery (LS), and open surgery (OS). RAS was defined as radical resection of pCCA performed using a robotic platform, such as the da Vinci system, either fully robot-led or with robotic assistance for key procedural steps. LS was defined as radical resection performed entirely laparoscopically or predominantly laparoscopically with minimal adjunctive open steps. OS was defined as conventional open radical resection through laparotomy. Direct comparisons could include LS versus OS, RAS versus OS, RAS versus LS, or multi-arm comparisons involving all three approaches.
3. Study Design: Comparative clinical studies were eligible, including prospective or retrospective cohort studies, case-control studies, and propensity-score matched or otherwise adjusted comparative studies. Randomized controlled trials, if available, were also eligible.
4. Outcomes: Perioperative efficacy and safety outcomes were considered the primary outcome domains. These included operative time, intraoperative blood loss, intraoperative transfusion rate, length of hospital stay, hospitalization costs, and major complications defined as Clavien–Dindo grade ≥ III. Secondary outcomes included R0 resection rate, perioperative changes in liver function and nutrition-related biochemical indices, and fixed-time survival outcomes, including 1- and 2-year overall survival. Studies reporting at least one eligible outcome were included.

#### 2.2.2 Exclusion criteria

1. Duplicate reports from the same trial or patient cohort; only the most complete and up-to-date study was included.
2. Studies that did not specifically focus on pCCA or included mixed populations (e.g., intrahepatic cholangiocarcinoma, gallbladder cancer) without extractable pCCA-specific data.
3. Non-comparative studies, case reports, small case series without a comparator group, reviews, editorials, conference abstracts without sufficient data, animal studies, or *in vitro* studies
4. Studies were also excluded when the anatomical definition of the tumor location was unclear and could not be confidently mapped to the prespecified perihilar definition.

### 2.3 Data extraction and quality assessment

Data extraction was performed independently by two investigators using a standardized extraction form, and disagreements were resolved through discussion with a third reviewer. For each eligible study, we extracted general study characteristics, patient characteristics, study design, sample size, surgical approaches compared, details of the intervention and comparator groups, follow-up duration, and outcome data. For direct pairwise comparisons, the number of patients in each surgical group was extracted separately to support the primary pairwise meta-analyses.

For continuous outcomes, group-specific means and standard deviations (SDs) were preferentially extracted. When data were reported as medians with ranges or interquartile ranges, they were converted to means and SDs using established methods when appropriate. For liver function and nutrition-related biochemical indices, postoperative–preoperative changes were extracted as mean changes with corresponding SDs whenever available. When only baseline and postoperative values were reported, the mean change was calculated as the difference between the two time points, and the SD of the change was estimated using the following formula:

$$SDchange=\sqrt{\mathrm{Baseline}\;SD^{2}+\mathrm{Endpoint}\;SD^{2}-2R\cdot \mathrm{Baseline}\;\mathrm{SD}\cdot \mathrm{Endpoint}\;\mathrm{SD}}$$

where R represents the assumed correlation coefficient between baseline and postoperative measurements. For dichotomous outcomes, the numbers of events and total sample sizes in each group were extracted. For survival outcomes, the numbers of patients alive and the total numbers of evaluable patients at 1 and 2 years were extracted separately for each surgical group. Time-to-event survival parameters, including overall survival hazard ratios, disease-free survival, recurrence-free survival, and Kaplan–Meier survival estimates, were also sought but were not sufficiently reported for quantitative synthesis. Hazard ratios with corresponding 95% confidence intervals were preferentially sought for time-to-event outcomes. However, because adjusted hazard ratios and the information required for their reconstruction were reported by too few studies, a pooled time-to-event survival analysis was not performed. When outcome definitions or reported statistics were unclear, attempts were made to contact the corresponding authors for clarification.

Because the included evidence was expected to be predominantly observational, we also extracted information on confounding control, including propensity-score matching, case matching, multivariable adjustment, or other methods used to improve comparability between surgical groups. In addition, clinically important variables that could influence treatment allocation and surgical outcomes in pCCA were descriptively extracted, including tumor extent or Bismuth classification, vascular involvement, extent of hepatectomy, need for biliary or vascular resection and reconstruction, overall case complexity, surgeon experience, learning-curve status, and institutional volume or expertise. We additionally recorded whether the included studies reported explicit eligibility criteria, contraindications, or institutional algorithms for selecting LS or RAS rather than OS. Reported selection-related factors were categorized into anatomical factors (including tumor extent, Bismuth classification, vascular involvement, and anticipated hepatectomy extent), technical factors (including reconstruction requirements and expected feasibility of minimally invasive procedures), and expertise-related factors (including surgeon experience and institutional volume). Where reported, selection-related factors included tumor extent, vascular involvement, future liver remnant, anticipated hepatectomy and reconstruction requirements, patient fitness, surgeon experience, and institutional expertise. Because these criteria were expected to be heterogeneous and primarily qualitative, they were synthesized descriptively rather than quantitatively.

To specifically assess operative extent as a potential effect modifier, we extracted group-specific data on vascular resection or reconstruction, isolated bile duct resection, minor hepatectomy, major or extended hepatectomy, caudate lobectomy, and biliary reconstruction. Isolated bile duct resection was defined as bile duct resection without formal hepatic resection. Minor hepatectomy was defined as resection of fewer than three Couinaud segments, whereas major hepatectomy was defined as resection of three or more segments; caudate lobectomy was recorded separately. For studies reporting propensity score-matched cohorts, matched data were preferentially extracted. Only values directly reported in the original studies were included. Values requiring calculation or inference from other reported data were recorded as NR.

Methodological quality was assessed independently by two investigators using the Newcastle–Ottawa Scale (NOS), which evaluates study quality across the domains of selection, comparability, and outcome assessment. NOS assessments were used to describe the quality of the direct comparative evidence and to inform restricted sensitivity analyses limited to higher-quality studies where feasible. Because key clinical effect modifiers were incompletely and inconsistently reported across studies, they could not be uniformly incorporated into quantitative adjustment or meta-regression. Therefore, these variables were used primarily for descriptive assessment of residual confounding, clinical heterogeneity, and the plausibility of indirect comparisons in the exploratory network meta-analysis.

Detailed NOS assessments are provided in Supplementary Table S3. Study-level summaries of tumor- and procedure-related effect modifiers, including operative extent and group-specific Bismuth–Corlette distributions, are presented in Supplementary Tables S4A, B. Information on confounding control, patient-selection criteria, contraindications, conversion criteria, surgical-approach allocation, and surgeon or institutional experience is summarized in Supplementary Table S5. Extracted study-level outcome data are provided in Supplementary Tables S6–S8.

### 2.4 Statistical analysis

Statistical analyses were performed using Stata 17.0 MP (11). The primary quantitative synthesis was based on direct pairwise meta-analyses of head-to-head comparisons. Continuous outcomes were summarized as mean differences (MDs) when measured on the same scale, and dichotomous outcomes were summarized as odds ratios (ORs). All effect estimates were reported with 95% confidence intervals (CIs) (12–14). For dichotomous outcomes with zero events or zero non-events in one treatment arm, a continuity correction was applied to avoid non-estimable ORs. One- and two-year overall survival rates were analyzed as separate fixed-time dichotomous outcomes using odds ratios with 95% confidence intervals when sufficient data were available. However, these analyses were not considered equivalent to conventional time-to-event survival analyses because they do not account for censoring, differences in follow-up duration, or the dynamic nature of survival outcomes. Although hazard ratios were sought whenever available, adjusted hazard ratios, Kaplan–Meier curves, and sufficient follow-up information were inconsistently reported across studies; therefore, pooled time-to-event survival analyses were not performed.

Separate direct pairwise analyses were conducted for LS versus OS and RAS versus OS when at least two studies reported the same outcome. One eligible study directly compared RAS with LS. Because only one RAS–LS study was available, its results were presented as study-specific effect estimates with 95% CIs and were not quantitatively pooled. Similarly, when only one study was available for any other outcome and comparison, the study-specific result was reported without meta-analysis or assessment of between-study heterogeneity. Direct pairwise evidence was considered the primary basis for clinical interpretation.

Between-study heterogeneity was assessed using the I^2^ statistic, *τ*^2^, Cochran's *Q* test, and the corresponding *P* value. Given the expected clinical and methodological heterogeneity across studies, including differences in tumor extent, operative complexity, reconstruction requirements, study design, and institutional experience, random-effects models were preferentially used for the primary pairwise meta-analyses. Fixed-effect models were considered only when the number of studies was limited and statistical heterogeneity was negligible. Forest plots for the primary pairwise meta-analyses are presented in Supplementary Figures S1-S4.

To assess the robustness of the primary direct evidence, restricted sensitivity analyses including only studies with Newcastle–Ottawa Scale scores of ≥7 were performed when at least two eligible studies remained available for the relevant comparison and outcome. These analyses were used to evaluate whether the main findings were maintained after excluding studies with lower methodological quality and are summarized in Supplementary Table S9. Leave-one-out sensitivity analyses were additionally performed for pooled pairwise analyses by sequentially omitting one study and recalculating the summary estimate. Leave-one-out analyses were not performed for comparisons supported by only one study. The corresponding sensitivity analysis plots are shown in Supplementary Figures S7–S10.

Potential publication bias and small-study effects were assessed using conventional funnel plots for the primary pairwise meta-analyses when a sufficient number of studies was available. Funnel plots were interpreted cautiously when fewer than 10 studies contributed to a given outcome or comparison. Funnel plots for perioperative and hospitalization-related outcomes are presented in Supplementary Figure S5, and those for oncological, biochemical, and survival outcomes are presented in Supplementary Figure S6. Funnel plots were not generated for single-study comparisons.

Because the overall evidence network remained sparse and unevenly distributed, with most studies comparing LS or RAS with OS, the network meta-analysis was performed only as a supplementary exploratory analysis to characterize the broader comparative evidence structure among RAS, LS, and OS. One small retrospective study provided a direct RAS–LS comparison. Therefore, closed evidence loops were available only for outcomes reported across all three pairwise comparisons, whereas outcomes without direct RAS–LS data retained an open or predominantly star-shaped network structure centered on OS. Network meta-analyses were conducted using random-effects models with restricted maximum likelihood estimation (15, 16). The outcome-specific evidence geometry is presented in Supplementary Figure S15, in which node size reflects the total number of participants receiving each surgical approach and line thickness reflects the number of studies contributing to each direct comparison (17). Given that the RAS–LS edge was supported by only one study, all network-derived estimates involving this comparison were interpreted as exploratory and hypothesis-generating.

The exploratory network analyses were conducted under the consistency assumption. Formal inconsistency assessment was not possible for outcome networks without a closed loop. For outcomes in which a triangular loop was present, the loop relied on only one small direct RAS–LS study; therefore, any statistical assessment of inconsistency would have had very limited power and was not considered sufficiently reliable for confirmatory interpretation. In addition, clinically important effect modifiers—including tumor extent, Bismuth classification, vascular involvement, isolated bile duct resection, major or extended hepatectomy, caudate lobectomy, biliary or vascular reconstruction, overall case complexity, surgeon experience, learning-curve status, and institutional surgical volume—were incompletely and inconsistently reported across studies. Consequently, the transitivity assumption could not be adequately verified. Network-derived direct and indirect estimates were therefore interpreted only as exploratory and were not used as the principal basis for clinical conclusions.

Exploratory leave-one-out sensitivity analyses were also conducted for network-derived estimates by sequentially omitting one study at a time and recalculating the corresponding network estimates. These numerical results are summarized in Supplementary Tables S14-S21. Because these analyses were based on the exploratory network framework, they were considered supplementary and were not used to validate the transitivity assumption or to support confirmatory inference.

Treatment ranking metrics, including the surface under the cumulative ranking curve (SUCRA), probability of being the best treatment, and mean rank values, were calculated only as exploratory supplementary information (17, 18). Rankings were interpreted cautiously because they may be unstable when evidence is sparse, imprecise, heterogeneous, or unevenly distributed and were not regarded as evidence of clinical superiority. Comparison-adjusted funnel plots were generated for the network analysis only where technically feasible. These plots were interpreted cautiously because most outcome networks included few studies, the evidence was unevenly distributed across comparisons, and the RAS–LS edge was supported by only one study. The exploratory network funnel plots are presented in Supplementary Figure S11.

Exploratory univariable network meta-regression analyses using selected study-level covariates, including mean age and male proportion, were performed where feasible. Given the small number of studies, observational nature of the included evidence, use of study-level rather than patient-level covariates, and risk of ecological bias, these meta-regression results were considered hypothesis-generating only and were not used to validate the transitivity assumption or to support confirmatory inference. The results of exploratory meta-regression analyses are summarized in Supplementary Tables S12, S13.

### 2.5 GRADE assessment

The certainty of evidence was assessed primarily for the direct pairwise comparisons using the GRADE framework (19, 20). Because all included studies were non-randomized comparative studies, the certainty of the direct evidence was initially rated as low and was further downgraded when concerns were identified regarding risk of bias, inconsistency, indirectness, imprecision, or publication bias. Particular attention was paid to confounding by indication, residual confounding, incomplete adjustment for baseline differences, sparse data, wide confidence intervals, and clinical heterogeneity related to tumor extent, Bismuth classification, vascular involvement, isolated bile duct resection, major or extended hepatectomy, caudate lobectomy, biliary or vascular reconstruction, case complexity, learning-curve status, and surgeon or institutional experience. For direct comparisons supported by only one study, the certainty of evidence was generally considered very low because of serious imprecision and the inability to assess between-study consistency.

For the exploratory network meta-analysis, CINeMA was used as a supplementary tool to assess confidence in network-derived estimates (21). The assessment considered within-study bias, reporting bias, indirectness, imprecision, heterogeneity, and incoherence. Formal assessment of incoherence was not possible for outcome networks without a closed evidence loop. For outcomes in which a closed loop was present, the direct RAS–LS edge was supported by only one small retrospective study; consequently, inconsistency assessments had limited statistical power and were not considered sufficiently reliable for confirmatory interpretation. In addition, clinically important effect modifiers were incompletely and inconsistently reported across comparisons, preventing adequate verification of the transitivity assumption. Network-derived confidence ratings were therefore interpreted cautiously and were not used as the primary basis for clinical conclusions.

Predefined thresholds for clinical importance were used to support judgments of imprecision where applicable (22), and are presented in Supplementary Table S10. Detailed certainty assessments for pooled direct comparisons, single-study direct estimates, and exploratory network estimates are provided in Supplementary Table S11.

## 3 Results

### 3.1 Literature selection and study characteristics

The initial literature search identified 248 records across the databases. After removal of duplicates and screening of titles and abstracts, 30 articles were selected for full-text review. Ultimately, 24 studies met the predefined inclusion and exclusion criteria and were included in the systematic review and quantitative synthesis (Figure 1) (8, 23–45).

**Figure 1:**
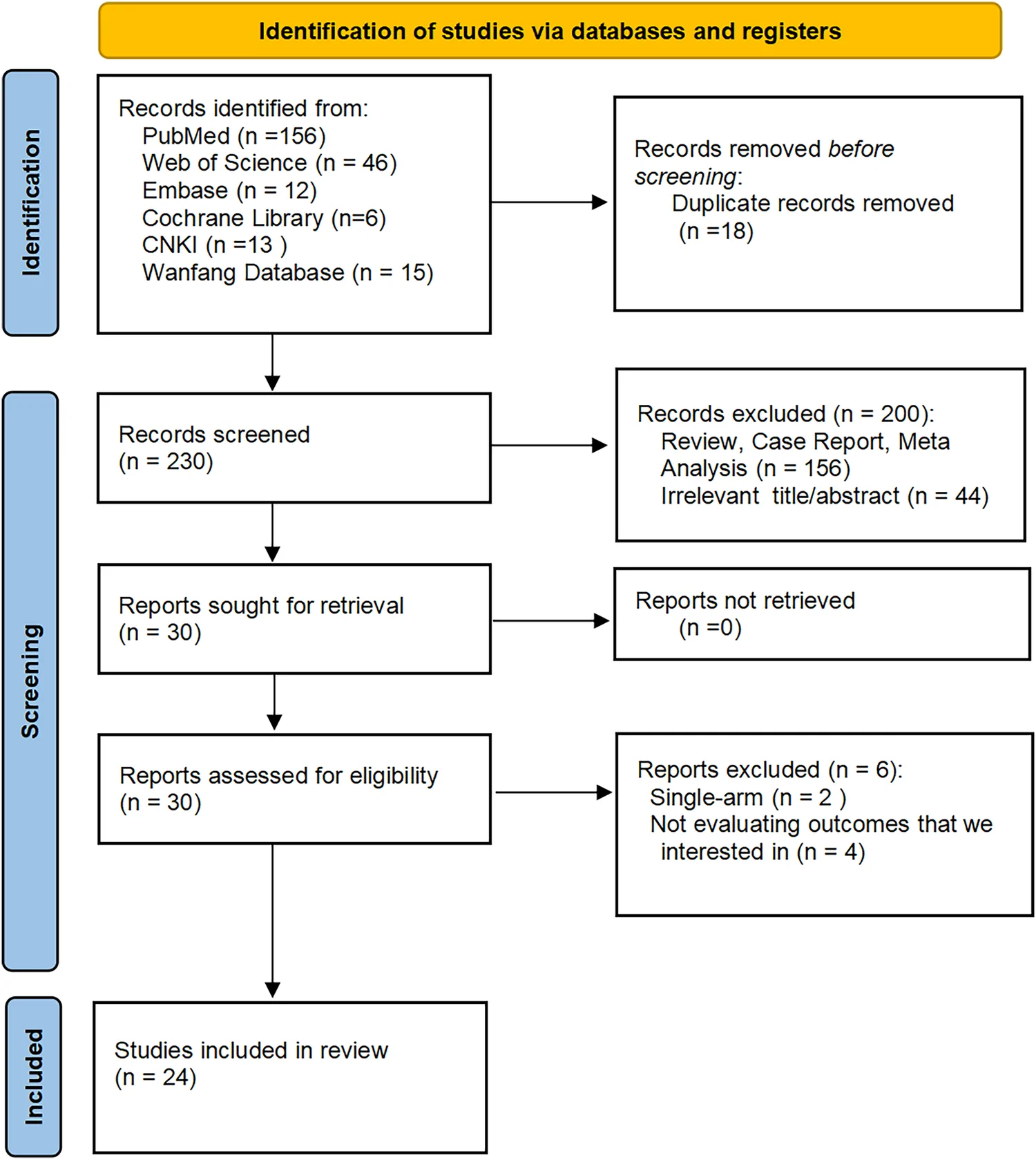
Flow diagram of literature search and study selection. Adapted from the PRISMA 2020 flow diagram template, licensed under CC BY 4.0.

A total of 1,598 patients with perihilar cholangiocarcinoma were included. Among the 24 studies, 18 directly compared laparoscopic surgery (LS) with open surgery (OS), five directly compared robot-assisted surgery (RAS) with OS, and one directly compared RAS with LS. Thus, the direct comparative evidence remained dominated by LS–OS and RAS–OS comparisons. The only direct RAS–LS evidence was derived from one small single-center retrospective case-control study and was therefore summarized descriptively using study-specific effect estimates rather than quantitatively pooled. Nevertheless, its inclusion provided a direct RAS–LS edge for the outcomes reported in that study and allowed a closed triangular evidence loop to be formed for selected outcome-specific networks.

Most studies were conducted in China, with additional studies from Italy, the United States, Portugal, Australia, and Russia. The evidence base consisted predominantly of single-center retrospective cohort or case-control studies. A smaller number of studies used propensity-score matching, case matching, multicenter retrospective designs, or prospective multicenter cohort designs. No randomized controlled trial was identified. Study populations generally comprised middle-aged to older adults, with reported mean ages ranging from approximately 55 to 67 years. Sex distributions were either approximately balanced or slightly male-predominant where reported. Follow-up duration and completeness varied substantially, and several studies did not provide sufficiently detailed long-term follow-up or time-to-event survival data.

Substantial clinical and methodological heterogeneity was present across the included studies. Reporting of tumor extent, Bismuth classification, vascular involvement, patient-selection criteria, isolated bile duct resection, major or extended hepatectomy, caudate lobectomy, biliary or vascular resection and reconstruction, conversion to open surgery, overall operative complexity, learning-curve status, surgeon experience, and institutional surgical volume was incomplete and inconsistent. These variables may have influenced both the selection of surgical approach and the observed perioperative and oncological outcomes. Because they were not reported consistently using comparable definitions and group-specific denominators, they could not be reliably incorporated into quantitative subgroup analyses or meta-regression and were therefore assessed primarily at the descriptive level.

Detailed baseline and study characteristics are presented in Table 1. Study-level information on tumor- and procedure-related effect modifiers, including operative extent and group-specific Bismuth–Corlette distributions, is provided in Supplementary Tables S4A, B. Information on confounding control, patient-selection criteria, contraindications, conversion criteria, surgical-approach allocation, and surgeon or institutional experience is summarized in Supplementary Table S5. Extracted perioperative, safety, oncological, survival, and biochemical outcome data are presented in Supplementary Tables S6–S8.

**Table 1:** Baseline characteristics of included studies.

No.	Study	Disease	Country	Follow-up (months)	Mean age (years)	Surgical approach (I vs. C)	Study design	Male	Female
1	Liu W 2023	pCCA	China (Wuhan)	NR	61.11	LS vs. OS	Single-center retrospective cohort	48.90%	51.10%
2	He YG 2022	pCCA	China (Chongqing)	OS 13.5 ± 9.30; LS 12 ± 5.37	63.00	LS vs. OS	Single-center retrospective cohort	47.90%	52.10%
3	Ma D 2022	pCCA	China (Shandong)	OS: 19.2; LS: 14.7	61.27	LS vs. OS	Single-center retrospective cohort	80.60%	19.40%
4	Ratti F 2020	pCCA	Italy (Milan)	LS: 14.7; OS: 19.2	62.33	LS vs. OS	Single-center retrospective cohort	52.10%	47.90%
5	Xiong F 2023	pCCA	China (Wuhan)	NR	55.70	LS vs. OS	Single-center retrospective cohort	50.00%	50.00%
6	Wang J 2023	Bismuth III-IV pCCA	China	OS:17.8 ± 12.3; LS ：23.9 ± 13.3	66.24	LS vs. OS	Single-center retrospective cohort	60.00%	40.00%
7	Yin Y 2024	pCCA	China	OS:11.42 ± 11.26 LS:9.37 ± 21.03	63.62	LS vs. OS	Single-center retrospective cohort	63.20%	36.80%
8	Zhang YH 2019	pCCA	China (Zhejiang)	NR	65.40	LS vs. OS	Single-center retrospective cohort	43.50%	56.50%
9	Chou S 2020	pCCA	China (Beijing)	22	62.72	RAS vs. OS	Single-center retrospective case-control	66.00%	34.00%
10	Xu Y 2016	pCCA	China (Beijing)	NR	57.81	RAS vs. OS	Single-center retrospective case-control	66.70%	33.30%
11	Jiang WX 2020	Bismuth I pCCA	China	NR	67.70	LS vs. OS	Single-center retrospective controlled study	52.80%	47.20%
12	Zhu H 2018	Bismuth I pCCA	China (Jiangxi)	6–18	60.05	LS vs. OS	Single-center retrospective controlled study	55.90%	44.10%
13	Duan C 2019	Bismuth I pCCA	China (Wuhan)	NR	60.65	LS vs. OS	Single-center retrospective controlled study	55.70%	42.30%
14	Gong JW 2014	pCCA	China	17.6 ± 0.8	57.57	LS vs. OS	NR	NR	NR
15	Chai W 2019	Bismuth IIIb pCCA	China	LS: 13; OS: 15	NR	LS vs. OS	Retrospective matched study	NR	NR
16	Sucandy I 2023	pCCA	USA, Portugal, and Italy	RAS:15; OS:NR	66.87	RAS vs. OS	Prospective multicenter cohort	NR	NR
17	Wang M 2023	pCCA	China	NR	62.82	LS vs. OS	Retrospective multicenter cohort	61.00%	39.00%
18	Liu H 2021	pCCA	China	NR	58.05	LS vs. OS	Retrospective cohort	NR	NR
19	Aniumuguo M 2022	pCCA	China	NR	53.33	LS vs. OS	Single-center retrospective cohort	NR	NR
20	Yao L 2021	Bismuth III-IV pCCA	China	All ≥12	61.06	LS vs. OS	Single-center retrospective cohort	NR	NR
21	Chen Z 2012	pCCA	China	NR	57.96	RAS vs. OS	Single-center retrospective cohort	69.23%	30.77%
22	Song W 2024	pCCA	China	NR	58.66	LS vs. OS	Retrospective case-control	66.70%	33.30%
23	Efanov M 2025	pCCA	Russia	NR	59.7	RAS vs. OS	Retrospective comparative cohort	64.30%	35.70%
24	Wang W 2025	pCCA	China	NR	64.51	RAS vs. LS	Single-center retrospective case-control study	66%	34%

The extent of surgical resection varied substantially across studies, as summarized in Supplementary Table S4A, while group-specific Bismuth–Corlette distributions are presented in Supplementary Table S4B. In the three studies restricted to Bismuth type I tumors, isolated bile duct resection was performed in all patients in both the minimally invasive and open groups, whereas major hepatectomy and caudate lobectomy were not performed. Conversely, several studies restricted to Bismuth type III–IV tumors reported major hepatectomy and caudate lobectomy in all or nearly all patients in both treatment groups. Among studies including mixed Bismuth classifications, the proportions of isolated bile duct resection, major hepatectomy, and caudate lobectomy varied considerably, and no consistent directional difference between minimally invasive and open surgery was observed. Although some minimally invasive cohorts had higher rates of isolated bile duct resection or lower rates of major hepatectomy and caudate lobectomy than their open comparators, this pattern was not uniform across studies. These findings suggest that operative extent was closely related to tumor distribution, Bismuth classification, patient selection, and institutional surgical strategy, in addition to the surgical approach itself.

Explicit indications and patient-selection strategies for minimally invasive surgery were rarely and inconsistently reported. Most studies did not describe a reproducible algorithm for choosing LS or RAS rather than OS, and few clearly specified technical contraindications to a minimally invasive approach. Although several studies reported Bismuth classification and selected indicators of tumor or operative complexity, information on vascular involvement, future liver remnant, anticipated reconstruction burden, patient fitness, surgeon experience, and institutional eligibility criteria was frequently incomplete. Therefore, the available studies did not permit identification of a consistent selection strategy or formal quantitative analysis of indications for minimally invasive pCCA resection.

### 3.2 Study quality assessment

The methodological quality of the 24 included studies was assessed using the Newcastle–Ottawa Scale (NOS), which evaluates three domains: selection of the study population, comparability between groups, and outcome assessment. NOS scores ranged from 6 to 9. Seventeen studies were classified as higher quality with NOS scores of ≥7, whereas seven studies had a score of 6. No study was rated as low quality according to the NOS criteria.

However, these NOS ratings should be interpreted cautiously because all included studies were non-randomized comparative studies and were therefore inherently susceptible to selection bias, confounding by indication, and residual confounding. The selection domain was generally well rated, reflecting clearly defined study populations and eligibility criteria. The outcome assessment domain was also generally acceptable, although some studies had limitations related to follow-up completeness, consistency of outcome assessment, or incomplete reporting of long-term outcomes.

The comparability domain represented the major methodological limitation. Only a subset of studies used propensity-score matching, case matching, multivariable adjustment, or other approaches to control for baseline differences between surgical groups. In many studies, clinically important factors such as tumor extent, Bismuth classification, vascular involvement, extent of hepatectomy, biliary or vascular reconstruction, case complexity, surgeon experience, and institutional expertise were incompletely reported or not adjusted for. Because these factors may strongly influence both treatment allocation and postoperative outcomes in pCCA surgery, the possibility of residual confounding remains substantial.

Therefore, although the included studies showed moderate to high NOS scores, the overall certainty of evidence remained limited. NOS assessments were used to describe study-level methodological quality and to inform restricted sensitivity analyses limited to studies with NOS ≥7, rather than to imply that the direct comparative evidence was free from bias. Detailed quality assessments are provided in Supplementary Table S3.

### 3.3 Direct evidence structure and exploratory network geometry

The direct comparative evidence was unevenly distributed across the three surgical approaches. Most included studies directly compared LS with OS, fewer studies compared RAS with OS, and only one small retrospective study directly compared RAS with LS. Therefore, the evidence base remained predominantly centered on OS as the common comparator. The direct RAS–LS evidence was presented as study-specific effect estimates and was not quantitatively pooled because only one eligible study was available.

The geometry of the evidence network varied across outcomes. For outcomes reported in all three direct comparisons—LS versus OS, RAS versus OS, and RAS versus LS—a nominal closed triangular evidence loop was present; however, the RAS–LS edge was supported by only one small retrospective study. However, the RAS–LS edge in each such loop was supported by only one small retrospective study. For outcomes not reported in the direct RAS–LS study, the network remained open or predominantly star-shaped, with OS serving as the common comparator.

Formal inconsistency assessment was not possible for outcome networks without a closed loop. For outcomes in which a triangular loop was present, the sparse evidence and reliance on a single direct RAS–LS study substantially limited the statistical power and reliability of global, local, or loop-specific inconsistency assessments. Consequently, agreement between direct and indirect evidence could not be evaluated robustly, and any network-derived comparison between RAS and LS remained highly uncertain.

The plausibility of the transitivity assumption was assessed descriptively by comparing reported clinical and methodological characteristics across the different direct comparisons. Although broad characteristics such as age and sex distribution appeared generally comparable in some studies, clinically important effect modifiers relevant to pCCA surgery were incompletely and inconsistently reported. These included tumor extent, Bismuth classification, vascular involvement, patient-selection criteria, isolated bile duct resection, major or extended hepatectomy, caudate lobectomy, biliary or vascular resection and reconstruction, operative complexity, learning-curve status, surgeon experience, and institutional surgical volume or expertise. Because these factors may influence both the selection of surgical approach and postoperative outcomes, the transitivity assumption could not be adequately verified, and residual intransitivity could not be excluded.

Accordingly, network-derived indirect comparisons and treatment rankings were interpreted only as exploratory and hypothesis-generating and were not used as the primary basis for clinical interpretation or conclusions, which relied principally on the available direct pairwise evidence. Study-level reporting of clinically relevant effect modifiers, including operative extent and group-specific Bismuth–Corlette distributions, is summarized in Supplementary Tables S4A, B, while information on confounding control, patient-selection criteria, surgical-approach allocation, and surgeon or institutional experience is provided in Supplementary Table S5. Outcome-specific network geometry is presented in Supplementary Figure S15. Exploratory league plots and treatment rankings are shown in Supplementary Figures S12–S14, and numerical results from the exploratory network leave-one-out sensitivity analyses are provided in Supplementary Tables S14–S21. Given the sparse and uneven evidence structure, particularly the reliance on a single study for the RAS–LS edge, these supplementary findings were not considered evidence of clinical superiority among the surgical approaches.

### 3.4 Patient selection criteria for minimally invasive surgery

Because patient selection is considered a critical determinant of outcomes in minimally invasive surgery for pCCA, we evaluated whether the included studies reported explicit criteria for selecting patients for LS or RAS rather than OS.

Explicit selection criteria for minimally invasive approaches were infrequently reported across the included studies. In most studies, the choice of LS or RAS appeared to be based on institutional preference, surgeon expertise, and perceived technical feasibility rather than predefined or prospectively validated selection algorithms. Although minimally invasive surgery was increasingly applied in pCCA, patients undergoing LS or RAS were generally considered highly selected cases, reflecting the requirement for favorable anatomical conditions and sufficient technical feasibility.

The reported selection-related factors mainly included tumor extent, Bismuth–Corlette classification, vascular involvement, anticipated extent of hepatectomy, feasibility of biliary reconstruction, patient condition, and surgeon or institutional experience. Several studies suggested that minimally invasive approaches were preferentially considered in patients without extensive vascular invasion, with technically feasible liver resection and reconstruction, and in centers with substantial experience in complex hepatobiliary surgery. However, the definitions of appropriate candidates varied considerably among studies, and detailed criteria for patient allocation were rarely standardized.

Importantly, no included study provided a universally applicable selection algorithm, validated indication criteria, or prospective evidence defining which patients with pCCA would derive the greatest benefit from minimally invasive surgery. Therefore, although minimally invasive approaches may be feasible in carefully selected patients, the current evidence remains insufficient to establish standardized indications or selection strategies for LS or RAS in pCCA.

### 3.5 Operative extent and surgical complexity

Given the complexity of radical resection for perihilar cholangiocarcinoma (pCCA), operative extent was considered an important clinical modifier that could influence perioperative outcomes. Therefore, procedure-related variables, including isolated bile duct resection, minor hepatectomy, major or extended hepatectomy, caudate lobectomy, and vascular or biliary reconstruction, were descriptively evaluated across included studies.

However, reporting of operative extent varied substantially among studies. Although several studies provided information regarding hepatectomy type and reconstruction procedures, others did not report these details separately according to surgical approach, limiting quantitative comparison.

Among studies reporting surgical extent, minimally invasive cohorts frequently represented highly selected patients and showed variable proportions of isolated bile duct resection or limited hepatectomy. In contrast, open surgery cohorts often included patients requiring more extensive procedures, including major hepatectomy, extended hepatectomy, caudate lobectomy, and complex reconstruction.

Several comparative studies demonstrated differences in operative extent between minimally invasive and open approaches, suggesting potential imbalance in surgical complexity between treatment groups (Supplementary Table S4A).

Because these procedure-related factors were inconsistently reported and could not be reliably incorporated into quantitative analyses, they were considered important sources of clinical heterogeneity and residual confounding when interpreting the comparative effectiveness of minimally invasive approaches.

### 3.6 Perioperative procedural and technical outcomes

#### 3.6.1 Operative time

Operative time was reported in 18 studies comparing LS with OS and in three studies comparing RAS with OS. No direct RAS versus LS comparison was available. Substantial heterogeneity was observed in both comparisons, particularly in the RAS versus OS comparison (LS vs. OS: I^2^ = 75.4%, *P* < 0.001; RAS vs. OS: I^2^ = 87.2%, *P* < 0.001); therefore, random-effects models were applied for both comparisons.

In the primary pairwise meta-analysis, both LS and RAS were associated with significantly longer operative time compared with OS (LS vs. OS: MD = 73.22 min, 95% CI: 57.49–88.96; RAS vs. OS: MD = 127.01 min, 95% CI: 63.79–190.26). These findings suggest that minimally invasive approaches require longer operative duration than OS, which may reflect greater technical complexity, reconstruction demands, and learning-curve effects in minimally invasive pCCA surgery. The corresponding forest plots are shown in Supplementary Figure S1.

The single RAS–LS study reported operative times of 414.5 ± 109.0 min for RAS and 389.0 ± 141.8 min for LS. Because only one direct study was available, no meta-analysis or heterogeneity assessment was performed, and the finding did not establish a clear difference between RAS and LS.

#### 3.6.2 Intraoperative blood loss

Intraoperative blood loss was reported in 18 studies comparing LS with OS and in three studies comparing RAS with OS. No direct RAS versus LS comparison was available. Between-study heterogeneity was substantial in the LS versus OS comparison and moderate in the RAS versus OS comparison (LS vs. OS: I^2^ = 76.8%, *P* < 0.001; RAS vs. OS: I^2^ = 60.3%, *P* = 0.056); therefore, random-effects models were applied. In the primary pairwise meta-analysis, both LS and RAS were associated with significantly lower intraoperative blood loss compared with OS (LS vs. OS: MD = −82.78 mL, 95% CI: −119.19 to −46.36; RAS vs. OS: MD = −91.47 mL, 95% CI: −175.84 to −7.11). These findings suggest that minimally invasive approaches may reduce intraoperative blood loss compared with OS, although the RAS estimate should be interpreted cautiously because it was based on only three direct studies. The corresponding forest plots are shown in Supplementary Figure S1.

In the single direct RAS–LS study, blood loss was lower in the RAS group than in the LS group. Because this evidence was derived from one small retrospective cohort, it was not pooled and should be interpreted cautiously.

#### 3.6.3 Intraoperative transfusion rate

Intraoperative transfusion rate was reported in nine studies comparing LS with OS and in two studies comparing RAS with OS. The single RAS–LS study did not report intraoperative transfusion outcomes. No significant statistical heterogeneity was observed in either direct comparison (LS vs. OS: I^2^ = 0.0%, *P* = 0.966; RAS vs. OS: I^2^ = 0.0%, *P* = 0.701); therefore, fixed-effects models were applied. In the primary pairwise meta-analysis, neither LS nor RAS was associated with a statistically significant difference in intraoperative transfusion rate compared with OS (LS vs. OS: OR = 0.86, 95% CI: 0.68–1.10; RAS vs. OS: OR = 0.95, 95% CI: 0.46–1.96). Although the point estimates were below 1, suggesting a possible trend toward reduced transfusion requirements with minimally invasive approaches, the confidence intervals crossed the null value. Therefore, the available direct evidence does not establish a clear transfusion-related advantage for either LS or RAS over OS. The corresponding forest plots are shown in Supplementary Figure S1.

### 3.7 Safety outcomes

#### 3.7.1 Clavien–dindo grade ≥ III complications

Major postoperative complications, defined as Clavien–Dindo grade ≥ III complications, were reported in eight studies comparing LS with OS and in four studies comparing RAS with OS. No significant statistical heterogeneity was observed in either direct comparison (LS vs. OS: I^2^ = 0.0%, *P* = 1.000; RAS vs. OS: I^2^ = 23%, *P* = 0.273); therefore, fixed-effects models were applied.

The pairwise meta-analysis showed no statistically significant difference in the odds of major postoperative complications between LS and OS (OR = 0.97, 95% CI 0.76–1.24) or between RAS and OS (OR = 1.31, 95% CI 0.73–2.33). Although the point estimate for RAS versus OS was greater than 1, the confidence interval crossed the null value and remained relatively wide, indicating uncertainty in both the direction and magnitude of the association. Therefore, the available direct comparative evidence does not demonstrate an increased or reduced risk of major postoperative complications with either LS or RAS compared with OS. The corresponding forest plots are presented in Supplementary Figure S2.

In the single direct RAS–LS study, major complications occurred in 7 of 28 patients undergoing RAS and 4 of 19 patients undergoing LS. The corresponding study-specific RR was 1.19 (95% CI: 0.42–3.35). Because the confidence interval was wide and only one study was available, no clear difference between RAS and LS could be established.

### 3.8 Resource utilization and economic outcomes

#### 3.8.1 Length of hospital stay

Length of hospital stay was reported in 18 LS–OS studies, four RAS–OS studies, and one direct RAS–LS study. Substantial heterogeneity was observed in both direct comparisons (LS vs. OS: I^2^ = 76.4%, *P* < 0.001; RAS vs. OS: I^2^ = 82.2%, *P* = 0.001); therefore, random-effects models were applied.

In the primary pairwise meta-analysis, laparoscopic surgery (LS) was associated with a significantly shorter hospital stay compared with open surgery (OS) (MD = −2.79 days, 95% CI −3.83 to −1.75). Robotic-assisted surgery (RAS) also yielded a significantly shorter hospital stay relative to OS (MD = −4.98 days, 95% CI −9.83 to −0.14). The corresponding forest plots are presented in Supplementary Figure 2.

In the single RAS–LS study, the mean length of hospital stay was 17.58 ± 12.30 days after RAS and 18.00 ± 5.61 days after LS. The study-specific estimate did not indicate a clear difference between the two minimally invasive approaches (MD = −0.42 days, 95% CI: −5.3 to 4.5).

#### 3.8.2 Hospitalization costs

Hospitalization cost was reported in four studies comparing LS with OS, one RAS–OS study, and one direct RAS–LS study. No significant statistical heterogeneity was observed among the included studies (I^2^ = 0.0%, *P* = 0.552); therefore, a fixed-effects model was applied. In the primary pairwise meta-analysis, LS was associated with significantly higher hospitalization cost compared with OS (MD = 0.82 × 10⁴ CNY, 95% CI: 0.26–1.37). This finding suggests that LS may increase direct hospitalization costs relative to OS, although the interpretation should be cautious because the analysis was based on a limited number of studies and cost estimates may be influenced by differences in hospital pricing systems, equipment use, perioperative management, and accounting methods. Only one study reported hospitalization costs for the RAS vs. OS comparison; therefore, meta-analysis was not performed. In this study, hospitalization costs tended to be higher in the RAS than in the OS, and the finding should be interpreted cautiously. The corresponding forest plots are shown in Supplementary Figure S2.

In the single RAS–LS study, hospitalization costs were 14.30 ± 4.76 × 10⁴ CNY for RAS and 12.75 ± 4.75 × 10⁴ CNY for LS. The study-specific estimate did not establish a clear difference between approaches.

### 3.9 Tumor resection quality and oncologic outcomes

#### 3.9.1 R0 resection rate

R0 resection was reported in twelve studies comparing LS with OS, four studies comparing RAS with OS, and one study directly comparing RAS with LS. No observed heterogeneity was detected in either the LS–OS comparison (I^2^ = 0.0%, *P* = 0.642) or the RAS–OS comparison (I^2^ = 0.0%, *P* = 0.584). Fixed-effect models were therefore applied. Because only one direct RAS–LS study was available, a conventional pairwise pooling could not be performed for this comparison.

The pairwise meta-analysis showed no statistically significant difference in the odds of R0 resection between LS and OS (OR = 1.30, 95% CI 0.87–1.93) or between RAS and OS (OR = 0.85, 95% CI 0.61–1.18). The point estimate was above 1 for LS versus OS but below 1 for RAS versus OS; however, both confidence intervals crossed the null value. Thus, the direct comparative evidence does not demonstrate an oncologic resection-quality advantage or disadvantage for either minimally invasive approach relative to OS. The corresponding forest plots are presented in Supplementary Figure S3.

In the single RAS–LS study, R0 resection was achieved in 24 of 28 RAS patients and 16 of 19 LS patients. The study-specific RR was 1.02 (95% CI: 0.68–1.52), providing no clear evidence of a difference between RAS and LS.

### 3.10 Liver function and nutritional biomarkers

#### 3.10.1 Changes in total bilirubin (T-BIL)

Changes in T-BIL was reported in six studies comparing LS with OS. No significant statistical heterogeneity was observed among the included studies (I^2^ = 34.3%, *P* = 0.179); therefore, a fixed-effects model was applied. In the primary pairwise meta-analysis, LS was associated with a greater reduction in T-BIL from pre- to post-intervention compared with OS (MD = −16.38 μmol/L, 95% CI: −23.02 to −9.74). This finding suggests that LS may be associated with a more favorable postoperative bilirubin profile than OS. Only one study reported the change in T-BIL for the RAS vs. OS comparison; therefore, meta-analysis was not performed. In this study, the reduction in T-BIL appeared slightly greater in the OS than in the RAS, but this finding should be interpreted cautiously. The corresponding forest plots are shown in Supplementary Supplementary Figure S3.

#### 3.10.2 Changes in alanine aminotransferase (ALT)

Changes in ALT was reported in five studies comparing LS with OS. Substantial heterogeneity was observed among the included studies (I^2^ = 88.2%, *P* < 0.001); therefore, a random-effects model was applied. In the primary pairwise meta-analysis, LS showed a tendency toward a smaller postoperative–preoperative change in ALT compared with OS, but the difference was not statistically significant (MD = −37.51 U/L, 95% CI: −83.96 to 8.95). This finding suggests that LS may be associated with less postoperative ALT fluctuation than OS, although the evidence remains uncertain. Only one study reported the change in ALT for the RAS vs. OS comparison; therefore, meta-analysis was not performed. In this study, the reduction in ALT was greater in the OS than in the RAS, and this single-study finding should be interpreted cautiously. The corresponding forest plots are shown in Supplementary Figure S3.

#### 3.10.3 Changes in serum albumin (ALB)

Changes in ALB was reported in five studies comparing LS with OS. Substantial heterogeneity was observed among the included studies (I^2^ = 88.9%, *P* < 0.001); therefore, a random-effects model was applied. In the primary pairwise meta-analysis, LS was not associated with a statistically significant difference in postoperative–preoperative ALB change compared with OS (MD = 1.76 g/L, 95% CI: −0.52 to 4.04). Although the point estimate suggested a smaller decline or more favorable change in ALB in the LS group, the confidence interval crossed the null value. Therefore, the current direct evidence is insufficient to establish a clear difference between LS and OS in postoperative ALB changes. Only one study reported the change in ALB for the RAS vs. OS comparison; therefore, meta-analysis was not performed. In this study, the decrease in ALB was smaller in the RAS than in the OS, and this single-study finding should be interpreted cautiously. The corresponding forest plots are shown in Supplementary Figure S3.

### 3.11 Fixed-time survival outcomes

Survival evidence was limited to fixed-time overall survival proportions. Most studies did not report adjusted hazard ratios, Kaplan–Meier-derived hazard estimates, or sufficiently detailed censoring information. Therefore, 1- and 2-year overall survival were analyzed separately as dichotomous outcomes, whereas a pooled time-to-event survival analysis was not performed.

#### 3.11.1 One-year overall survival

One-year overall survival was reported in six LS–OS studies, three RAS–OS studies, and one direct RAS–LS study. No significant statistical heterogeneity was observed in either direct comparison (LS vs. OS: I^2^ = 0.0%, *P* = 0.971; RAS vs. OS: I^2^ = 0.0%, *P* = 0.775); therefore, fixed-effects models were applied.

In the primary pairwise meta-analysis, neither LS nor RAS was associated with a statistically significant difference in 1-year overall survival compared with OS (LS vs. OS: OR = 1.00, 95% CI: 0.57–1.75; RAS vs. OS: OR = 1.19, 95% CI: 0.71–1.98). The LS versus OS estimate suggested nearly identical 1-year survival between the two approaches, while the RAS versus OS estimate showed a non-significant trend favoring RAS. Overall, the available direct evidence does not establish a clear short-term survival advantage for either minimally invasive approach over OS. The corresponding forest plots are shown in Supplementary Figure S4.

The single RAS–LS study reported one-year overall survival rates of 63.5% after RAS and 68.1% after LS. Because these were fixed-time survival estimates from one retrospective study, they were summarized descriptively and were not pooled.

#### 3.11.2 Two-year overall survival

Two-year overall survival was reported in five LS–OS studies, three RAS–OS studies, and one direct RAS–LS study. No significant statistical heterogeneity was observed in either direct comparison (LS vs. OS: I^2^ = 0.0%, *P* = 0.544; RAS vs. OS: I^2^ = 1.0%, *P* = 0.364); therefore, fixed-effects models were applied.

In the primary pairwise meta-analysis, neither LS nor RAS was associated with a statistically significant difference in 2-year overall survival compared with OS (LS vs. OS: OR = 0.86, 95% CI: 0.58–1.27; RAS vs. OS: OR = 1.23, 95% CI: 0.79–1.91). The LS versus OS estimate showed no survival advantage for LS, while the RAS versus OS estimate suggested a non-significant trend favoring RAS. Overall, the available direct evidence does not support a clear 2-year survival advantage for either minimally invasive approach over OS. The corresponding forest plots are shown in Supplementary Figure S4.

In the single RAS–LS study, the reported 2-year overall survival rates were 42.0% after RAS and 40.0% after LS. This finding was not pooled and should not be interpreted as evidence of survival equivalence.

### 3.12 Sensitivity analyses, exploratory meta-regression, and small-study effects

The primary direct pairwise meta-analyses are summarized in Table 2. Compared with OS, both LS and RAS were associated with longer operative time, lower intraoperative blood loss, and shorter length of hospital stay. LS was additionally associated with higher hospitalization costs than OS. No clear differences were observed in intraoperative transfusion rate, R0 resection rate, 1-year overall survival, or 2-year overall survival. For major complications, neither LS nor RAS differed significantly from OS in the primary pairwise analyses. The RAS–OS point estimate was greater than 1, but its confidence interval crossed the null value; this estimate was based on a limited number of retrospective studies and was rated as very-low-certainty evidence. Direct RAS–LS evidence was available from only one small retrospective study; therefore, its outcome-specific estimates were reported descriptively without quantitative pooling. Evidence for biochemical outcomes, including changes in T-BIL, ALT, and ALB, was limited and heterogeneous and should be interpreted cautiously.

**Table 2:** Summary of pooled effects and heterogeneity in pairwise meta-analyses.

Outcome	Comparison	Effect measure	Pooled effect	Lower 95% CI	Upper 95% CI	I^2^ (%)
Operative time (min)	LS vs. OS	MD	73.22	57.49	88.96	75.4
Operative time (min)	RAS vs. OS	MD	127.01	63.74	190.26	87.2
Intraoperative blood loss (mL)	LS vs. OS	MD	−82.78	−119.19	−46.36	76.8
Intraoperative blood loss (mL)	RAS vs. OS	MD	−91.47	−175.84	−7.11	60.3
Intraoperative transfusion rate	LS vs. OS	OR	0.86	0.68	1.1	0
Intraoperative transfusion rate	RAS vs. OS	OR	0.95	0.46	1.96	0
Major complications (Clavien–Dindo grade ≥ III)	LS vs. OS	OR	0.97	0.76	1.24	0
Major complications (Clavien–Dindo grade ≥ III)	RAS vs. OS	OR	1.31	0.73	2.33	23.0
Length of hospital stay (days)	LS vs. OS	MD	−2.79	−3.83	−1.75	76.4
Length of hospital stay (days)	RAS vs. OS	MD	−4.98	−9.83	−0.14	82.2
Hospitalization costs (×10⁴ CNY)	LS vs. OS	MD	0.82	0.26	1.37	0
Hospitalization costs (×10⁴ CNY)	RAS vs. OS	MD	Not pooled	NA	NA	NA
R0 resection rate	LS vs. OS	OR	1.3	0.87	1.93	0
R0 resection rate	RAS vs. OS	OR	0.85	0.61	1.18	0
Change in T-BIL (*μ*mol/L)	LS vs. OS	MD	−16.38	−23.02	−9.74	34.3
Change in T-BIL (μmol/L)	RAS vs. OS	MD	Not pooled	NA	NA	NA
Change in ALT (U/L)	LS vs. OS	MD	−37.51	−83.96	8.95	88.2
Change in ALT (U/L)	RAS vs. OS	MD	Not pooled	NA	NA	NA
Change in ALB (g/L)	LS vs. OS	MD	1.76	−0.52	4.04	88.9
Change in ALB (g/L)	RAS vs. OS	MD	Not pooled	NA	NA	NA
1-year overall survival	LS vs. OS	OR	1	0.57	1.75	0
1-year overall survival	RAS vs. OS	OR	1.19	0.71	1.98	0
2-year overall survival	LS vs. OS	OR	0.86	0.58	1.27	0
2-year overall survival	RAS vs. OS	OR	1.23	0.79	1.91	1

NA, not applicable. Meta-analysis was not performed when only one study was available for a given outcome and comparison.

Restricted sensitivity analyses including only studies with Newcastle–Ottawa Scale scores of ≥7 were performed when at least two eligible studies remained available for the relevant comparison and outcome. The results are summarized in Supplementary Table S9. Overall, the principal findings for operative time, intraoperative blood loss, and length of hospital stay were broadly maintained after restriction to higher-quality studies. Both LS and RAS remained associated with longer operative time than OS, and LS remained associated with lower intraoperative blood loss and shorter hospital stay. RAS also remained associated with shorter hospital stay, whereas its effects on intraoperative blood loss and transfusion rate remained statistically uncertain. No clear difference in R0 resection rate was observed in either the LS–OS or RAS–OS comparison.

For major complications, the restricted LS–OS analysis remained consistent with the primary analysis and did not demonstrate a clear difference between approaches. The RAS–OS estimate was sensitive to study inclusion and changed materially after restriction according to methodological quality. This instability, together with the small number of contributing studies and differences in case complexity and operative extent, indicates that the comparative safety estimate for RAS should not be regarded as robust. The restricted sensitivity analysis was therefore interpreted as an assessment of result stability rather than evidence that either RAS or OS was superior. No restricted meta-analysis was performed for direct RAS–LS comparisons because only one study was available.

Leave-one-out sensitivity analyses indicated that the main perioperative findings in the LS versus OS comparison, including operative time, intraoperative blood loss, length of hospital stay, and hospitalization cost, were generally robust to the exclusion of individual studies. The non-significant findings for intraoperative transfusion rate, major complications, R0 resection rate, and survival outcomes were also largely unchanged. By contrast, liver function-related outcomes, particularly changes in T-BIL and ALT, showed greater variability after single-study exclusion. In the RAS versus OS comparison, sensitivity analyses were constrained by the small number of studies, and several estimates showed noticeable fluctuation with wide confidence intervals. Therefore, the RAS-related results and liver function-related outcomes should be interpreted cautiously. Leave-one-out analyses were not performed for comparisons supported by only one study, including the direct RAS–LS comparison and selected RAS-related cost and biochemical outcomes. The corresponding sensitivity analysis plots are shown in Supplementary Figures S7–S10.

Conventional funnel plots were generated for pooled direct comparisons where technically appropriate and are presented in Supplementary Figures S5 and S6. In the LS–OS analyses, some asymmetry was observed for operative time, intraoperative blood loss, and R0 resection rate, indicating that small-study effects or publication bias could not be excluded. The funnel plot for length of hospital stay appeared relatively symmetrical, although dispersion between studies remained evident. For hospitalization costs, biochemical indices, and survival outcomes, the small number of contributing studies precluded reliable assessment of funnel-plot asymmetry. Most RAS–OS outcomes were supported by only a small number of studies (generally ≤5 studies); therefore, their funnel plots had limited interpretability and no firm conclusion regarding publication bias could be drawn. Funnel plots were not generated or interpreted for single-study comparisons, including the direct RAS–LS evidence.

Exploratory leave-one-out analyses were additionally conducted for network-derived estimates by sequentially excluding individual studies and recalculating the corresponding estimates where the network remained connected and estimable. Numerical results are summarized in Supplementary Tables S14–S21. These analyses were undertaken solely to explore the influence of individual studies on the supplementary network results and were not used to validate the consistency or transitivity assumptions. The direction of most network estimates was broadly maintained, but estimates involving RAS were frequently unstable because of the limited number and small sample sizes of the contributing studies. For outcome networks in which the only direct RAS–LS study was omitted, the RAS–LS edge and corresponding closed loop were lost; the remaining RAS–LS estimate was then based entirely on indirect evidence through OS. Results from these disconnected or structurally altered networks were interpreted with particular caution.

Study-level clinical characteristics may help explain some of the observed instability. Information on tumor extent, vascular involvement, isolated bile duct resection, minor and major or extended hepatectomy, caudate lobectomy, biliary and vascular reconstruction, and overall operative complexity is summarized in Supplementary Table S4A, while group-specific Bismuth–Corlette distributions are presented in Supplementary Table S4B. Information on adjustment for confounding, patient-selection criteria, contraindications, conversion criteria, surgical-approach allocation, surgeon experience, learning-curve status, and institutional expertise is summarized in Supplementary Table S5. Several LS–OS studies were single-center retrospective cohorts in which patients selected for LS may have had less extensive disease or lower reconstruction requirements than those selected for OS. Likewise, RAS–OS studies included both early institutional robotic experiences and more recent expert-center cohorts undertaking major hepatectomy, caudate lobectomy, and selected vascular reconstruction. Differences in operative extent, Bismuth classification, institutional volume, technical maturity, case selection, and perioperative pathways may therefore have contributed to between-study variation and residual confounding.

Exploratory univariable network meta-regression analyses were conducted using selected aggregate study-level covariates, including mean age and the proportion of male participants, where sufficient data were available. The results are presented in Supplementary Tables S12, S13. No consistent pattern of effect modification was observed across outcomes. Isolated statistically significant associations, particularly for selected biochemical outcomes, were interpreted cautiously because of the small number of studies, observational study designs, use of aggregate rather than patient-level data, ecological bias, model instability, multiple testing, and the possibility of chance findings. Clinically important variables such as institutional surgical volume, patient-selection criteria, major hepatectomy, caudate lobectomy, isolated bile duct resection, and vascular reconstruction could not be reliably examined because they were incompletely and inconsistently reported. The meta-regression findings were therefore considered hypothesis-generating and were not used to validate transitivity or support confirmatory conclusions.

Exploratory comparison-adjusted funnel plots for the network meta-analysis are presented in Supplementary Figure S11. Their interpretation was limited by the small number of studies, uneven distribution of evidence across comparisons, outcome-specific network geometry, and the fact that the direct RAS–LS edge was supported by only one study. Some outcome networks retained an open or predominantly star-shaped structure centered on OS, whereas selected outcomes formed a triangular loop through the single RAS–LS study. Accordingly, network-based assessments of small-study effects were considered supplementary and were not used as the primary basis for judging publication bias.

Exploratory treatment-ranking and league plots are presented in Supplementary Figures S12–S14. For outcomes with direct evidence across all three comparisons, the closed loop relied on only one small RAS–LS study; for other outcomes, the network remained open or predominantly centered on OS. Because the evidence was sparse, imprecise, clinically heterogeneous, and unevenly distributed, treatment rankings were considered unstable and hypothesis-generating. They were not interpreted as evidence of clinical superiority and were not used to determine the primary conclusions.

### 3.13 Certainty of evidence assessment

The certainty of evidence was assessed primarily using GRADE for direct pairwise comparisons, with CINeMA applied as a supplementary assessment of exploratory network estimates (Supplementary Table S11). Overall, the certainty was low or very low because all included studies were non-randomized, with risks of selection bias, residual confounding, sparse data, wide confidence intervals, and incomplete reporting of important clinical effect modifiers, including tumor extent, operative complexity, extent of hepatectomy, caudate lobectomy, reconstruction requirements, and institutional experience. Direct evidence suggested lower blood loss and shorter hospital stay but longer operative time with minimally invasive approaches; no clear differences were observed in transfusion, R0 resection, or 1- and 2-year survival. Neither LS nor RAS differed clearly from OS in major complications. Although the point estimate for RAS versus OS was greater than 1, the confidence interval crossed the null value, and the evidence was of very low certainty. Direct RAS–LS evidence was limited to one small retrospective study. Network confidence was further reduced by the sparse and uneven evidence structure, outcome-specific availability of closed loops, limited power to assess incoherence, and inability to adequately verify transitivity. Accordingly, network estimates and treatment rankings were considered exploratory and were not used as the primary basis for clinical conclusions.

## 4 Discussion

### 4.1 Key findings

This review prioritized direct pairwise evidence when comparing RAS, LS, and OS for pCCA, while retaining network-derived estimates as exploratory supplementary evidence. A total of 24 comparative studies involving 1,598 patients were included. Most direct evidence was derived from LS–OS and RAS–OS comparisons, whereas only one small retrospective study directly compared RAS with LS. Therefore, the principal clinical interpretation was based on direct pairwise evidence. The single-study RAS–LS estimates and network-derived indirect comparisons were considered exploratory and were not regarded as confirmatory evidence.

The direct pairwise analyses suggested that minimally invasive approaches may provide selected short-term perioperative benefits compared with OS. Both LS and RAS were associated with lower intraoperative blood loss and shorter hospital stay, but also with longer operative time. These findings may reflect reduced surgical trauma and enhanced postoperative recovery, while also demonstrating the technical complexity and prolonged procedural duration associated with minimally invasive pCCA resection. LS was additionally associated with higher hospitalization costs than OS. Nevertheless, these perioperative findings should be interpreted in the context of possible differences in patient selection, operative extent, perioperative pathways, and institutional experience (51).

No clear difference in intraoperative transfusion rate was observed between either minimally invasive approach and OS. Neither LS nor RAS demonstrated a statistically significant difference in major complications compared with OS in the primary analyses. Although the restricted sensitivity analysis suggested a possible reduction in major complications with RAS, this finding was unstable and should not be interpreted as evidence of superiority. For RAS versus OS, the point estimate was greater than 1, but the confidence interval was wide and crossed the null value. This estimate was derived from a limited number of retrospective studies and was rated as very-low-certainty evidence. Differences in tumor complexity, major or extended hepatectomy, caudate lobectomy, vascular resection and reconstruction, learning-curve status, and institutional case volume may have contributed to this finding. Accordingly, the available evidence does not permit a definitive conclusion regarding the comparative safety of RAS.

No clear differences were identified in R0 resection rate or fixed-time 1- and 2-year overall survival. However, the absence of statistically significant differences should not be interpreted as evidence of oncological or survival equivalence. The survival analyses were based primarily on unadjusted fixed-time survival proportions and did not fully account for censoring or differences in follow-up duration. Long-term survival evidence was sparse, follow-up and survival reporting were inconsistent, and most studies did not provide adjusted hazard ratios. Current evidence therefore remains insufficient to establish the long-term oncological equivalence or superiority of RAS, LS, or OS.

Evidence regarding liver function and nutrition-related biochemical outcomes was limited and heterogeneous. LS showed a significant difference in postoperative T-BIL change compared with OS, whereas no clear differences were observed for ALT or ALB. These findings may have been influenced by baseline jaundice severity, preoperative biliary drainage, liver functional reserve, extent of hepatectomy, biliary reconstruction, postoperative infection, nutritional status, and the timing of biochemical measurement. They should therefore be regarded as exploratory rather than clinically decisive outcomes.

The geometry of the exploratory network varied by outcome. Selected outcomes formed a triangular loop through the single direct RAS–LS study, whereas outcomes without direct RAS–LS data retained an open or predominantly star-shaped structure centered on OS. Because the RAS–LS edge was supported by only one small retrospective study and clinically important effect modifiers were incompletely reported, the transitivity assumption could not be adequately verified and inconsistency could not be assessed reliably. Network estimates and treatment rankings should therefore be regarded as hypothesis-generating and should not be interpreted as evidence of comparative superiority.

Overall, the certainty of evidence was low or very low across most outcomes. The findings support the technical feasibility of minimally invasive pCCA resection in carefully selected patients at experienced centers but are not sufficiently robust to justify practice-changing conclusions or standardized indications. Selection of the surgical approach should continue to be individualized according to tumor extent, vascular involvement, future liver remnant, anticipated hepatectomy and reconstruction requirements, patient condition, and surgeon and institutional expertise.

### 4.2 Interpretation of perioperative outcomes

The primary direct pairwise meta-analyses suggested that minimally invasive approaches may provide selected short-term perioperative advantages compared with OS, particularly reduced intraoperative blood loss. Both LS and RAS were associated with significantly lower blood loss than OS. Potential explanations include magnified visualization, improved identification of anatomical planes, precise vascular control, and meticulous tissue dissection and hemostasis (46–48).

However, these potential advantages should be interpreted together with the longer operative duration observed for minimally invasive approaches. Both LS and RAS were associated with significantly longer operative time than OS, which may reflect the technical complexity of minimally invasive pCCA surgery, including hilar dissection, vascular control, lymphadenectomy, and biliary-enteric reconstruction. Therefore, the benefits of minimally invasive surgery should not be considered independently from the complexity of the procedures performed.

Robotic systems may theoretically provide additional advantages over conventional laparoscopy through three-dimensional visualization, wristed instruments, tremor filtration, and improved dexterity in confined operative fields. These features may be particularly relevant during hilar dissection, biliary reconstruction, and vascular procedures (49, 50). Nevertheless, current comparative evidence regarding RAS remains limited. Only one retrospective study directly compared RAS with LS, and its outcome-specific estimates could not be quantitatively pooled. Therefore, whether the theoretical advantages of robotic platforms translate into clinically meaningful superiority remains uncertain.

For transfusion rate and major postoperative complications, no significant differences were demonstrated between minimally invasive approaches and OS. These outcomes are influenced by multiple factors beyond surgical access, including tumor extent, vascular involvement, future liver remnant, extent of hepatectomy, reconstruction requirements, perioperative management, and institutional experience. Consequently, the available evidence does not establish clear safety superiority of LS or RAS over OS.

### 4.3 Operative complexity, patient selection, and implications for minimally invasive surgery

Unlike many other abdominal procedures, minimally invasive surgery for pCCA represents a particularly challenging scenario because radical resection frequently requires major anatomical liver resection, caudate lobectomy, lymphadenectomy, and complex biliary reconstruction. Therefore, postoperative outcomes are determined not only by the surgical approach itself but also by tumor extent, operative complexity, reconstruction requirements, and institutional expertise.

An important unresolved issue in the current literature is the appropriate selection of patients for minimally invasive pCCA surgery. Although minimally invasive approaches have increasingly been reported for pCCA, explicit criteria defining indications, contraindications, or selection strategies for laparoscopic surgery (LS) or robot-assisted surgery (RAS) remain poorly described. Most studies appeared to select patients according to perceived technical feasibility, anatomical characteristics, and surgeon or institutional experience rather than standardized or prospectively validated algorithms.

Reported selection-related factors mainly included tumor extent, Bismuth–Corlette classification, vascular involvement, anticipated extent of hepatectomy, feasibility of biliary reconstruction, patient condition, and institutional expertise (57). However, the definitions of appropriate candidates varied considerably among studies, and no universally applicable selection algorithm or validated indication criteria have been established.

Operative extent represents another major source of potential confounding. Radical pCCA surgery is not a uniform procedure; it may range from isolated extrahepatic bile duct resection to major or extended hepatectomy combined with caudate lobectomy, vascular reconstruction, and complex biliary reconstruction. The concern that minimally invasive cohorts may include a higher proportion of isolated bile duct resections and fewer major liver resections or caudate lobectomies is clinically relevant. Although operative data extracted from the included studies demonstrated that this pattern was not completely consistent across all cohorts, differences in operative extent remained evident in some early or highly selected minimally invasive series.

Therefore, the observed perioperative advantages of minimally invasive approaches, including reduced blood loss and shorter hospital stay, should be interpreted cautiously. These benefits may partly reflect differences in patient selection, tumor complexity, and operative extent rather than solely the effect of the surgical access route itself. In particular, minimally invasive surgery may be preferentially applied to patients with favorable anatomical characteristics, limited vascular involvement, technically feasible reconstruction, and lower anticipated operative complexity, whereas open surgery may remain the preferred approach for patients requiring extensive hepatectomy, caudate lobectomy, vascular resection, or complex reconstruction.

Although we extracted clinically relevant variables related to patient selection and operative complexity, including tumor extent, vascular involvement, isolated bile duct resection, extent of hepatectomy, caudate lobectomy, reconstruction requirements, and surgeon or institutional experience, these factors were inconsistently reported and could not be quantitatively adjusted. Consequently, residual confounding related to surgical complexity remains an important limitation of current evidence.

Future multicenter prospective studies should establish standardized selection criteria incorporating anatomical factors, tumor characteristics, operative requirements, and institutional expertise to determine which patients with pCCA are most likely to benefit from minimally invasive approaches.

### 4.4 Long-term oncological outcomes and survival evidence

Oncological safety remains a central consideration when evaluating minimally invasive surgery for pCCA. In the present analysis, no clear differences were observed in R0 resection rate, 1-year overall survival, or 2-year overall survival between minimally invasive approaches and OS. However, these findings should not be interpreted as evidence of oncological equivalence.

The available survival evidence was limited primarily to fixed-time survival proportions, whereas conventional time-to-event analyses were not feasible because most studies did not provide adjusted hazard ratios, Kaplan–Meier curves, or sufficient follow-up information. Differences in follow-up duration, censoring patterns, tumor characteristics, adjuvant treatment, and baseline risk factors could therefore not be adequately accounted for.

Furthermore, long-term oncological outcomes are closely related to tumor biology and surgical radicality, including margin status, lymph node involvement, vascular invasion, and extent of resection. Without standardized adjustment for these factors, differences in survival outcomes cannot be confidently attributed to the surgical approach alone (55).

Therefore, current evidence remains insufficient to determine whether LS or RAS provides equivalent long-term oncological outcomes compared with OS. Future studies should incorporate standardized reporting of overall survival, disease-free survival, recurrence patterns, and adjusted time-to-event analyses with adequate follow-up.

### 4.5 Network evidence and clinical interpretation

The exploratory network meta-analysis was performed to characterize the broader comparative evidence structure among RAS, LS, and OS but was not intended to provide confirmatory treatment ranking.

The network geometry varied according to outcome. For outcomes reported across LS–OS, RAS–OS, and RAS–LS comparisons, a triangular evidence loop was formed through the single direct RAS–LS study. However, outcomes without direct RAS–LS data retained an open or predominantly star-shaped structure centered on OS.

Because the direct RAS–LS comparison was supported by only one small retrospective study, statistical assessment of inconsistency was limited and network-derived estimates remained highly dependent on indirect evidence. Furthermore, important effect modifiers, including tumor extent, Bismuth classification, operative extent, vascular involvement, reconstruction requirements, patient selection criteria, institutional volume, and surgeon experience, were incompletely reported.

Consequently, the transitivity assumption could not be adequately verified. Network estimates and treatment rankings should therefore be interpreted as exploratory and hypothesis-generating rather than evidence of comparative superiority.

### 4.6 Comparison with previous studies

Previous systematic reviews and meta-analyses comparing minimally invasive surgery with OS for pCCA have generally suggested that LS or RAS may reduce intraoperative blood loss and shorten postoperative hospital stay when performed in carefully selected patients at experienced centers. However, evidence regarding transfusion requirements, major morbidity, margin-negative resection, and long-term survival has remained uncertain because existing studies are largely retrospective and heterogeneous (56).

The present review provides an updated synthesis incorporating recently published comparative cohorts (52) and the limited direct evidence comparing RAS with LS. Consistent with previous reports (53, 54), minimally invasive approaches were associated with reduced blood loss and shorter hospital stay but required longer operative time. No clear differences were identified in transfusion rate, R0 resection rate, major complications, or fixed-time survival outcomes.

Compared with earlier studies, the current review places greater emphasis on the influence of operative complexity and patient selection. Earlier minimally invasive series frequently involved isolated bile duct resection or limited hepatic procedures, whereas recent expert-center studies have increasingly reported more complex resections, including major hepatectomy, caudate lobectomy, and selected vascular reconstruction. Pooling these clinically heterogeneous procedures under a single minimally invasive category may obscure important differences in surgical difficulty and oncological radicality.

Therefore, future comparative studies should avoid interpreting minimally invasive surgery as a uniform intervention and should instead account for tumor complexity, operative extent, and institutional expertise.

### 4.7 Strengths and limitations

This study has several strengths. First, the protocol was prospectively registered, and the review was conducted using systematic searching, independent study selection, standardized data extraction, and reporting procedures consistent with PRISMA recommendations. Second, direct head-to-head evidence was prioritized through separate pairwise analyses of LS versus OS and RAS versus OS, while direct RAS–LS evidence was presented descriptively because only one eligible study was available. Third, an exploratory network meta-analysis was performed to characterize the comparative evidence structure among RAS, LS, and OS; however, network estimates and treatment rankings were interpreted cautiously as supplementary rather than confirmatory evidence. Fourth, clinically relevant tumor-, procedure-, and center-related variables were systematically extracted, including Bismuth classification, vascular involvement, operative extent, isolated bile duct resection, major or extended hepatectomy, caudate lobectomy, reconstruction requirements, patient-selection criteria, surgeon experience, and institutional expertise.

Several limitations should nevertheless be acknowledged. First, all included studies were observational comparative studies, resulting in potential confounding by indication and residual selection bias. Although some studies applied propensity-score matching or other adjustment methods, important determinants of surgical selection were incompletely controlled.

Second, the evidence network remained sparse, particularly for RAS. Only one retrospective study directly compared RAS with LS, limiting confidence in comparisons between minimally invasive approaches.

Third, clinically important effect modifiers, including tumor extent, Bismuth classification, vascular involvement, operative extent, reconstruction requirements, institutional volume, and surgeon experience, were inconsistently reported. Therefore, the transitivity assumption for network analysis could not be adequately verified.

Fourth, long-term oncological outcomes were insufficiently reported. Survival analyses were limited by inconsistent follow-up, incomplete time-to-event information, and lack of adjusted hazard ratios.

Finally, the optimal indications for minimally invasive pCCA surgery remain undefined because standardized patient-selection criteria and prospective validation studies are lacking.

### 4.8 Conclusions

In this systematic review and direct pairwise meta-analysis of 24 comparative studies involving 1,598 patients, both LS and RAS were associated with lower intraoperative blood loss and shorter hospital stay compared with OS but required longer operative time. No clear differences were observed in transfusion rate, R0 resection rate, major complications, or fixed-time 1- and 2-year overall survival.

However, the interpretation of these findings is limited by sparse direct evidence, substantial clinical heterogeneity, incomplete reporting of operative extent, and potential confounding related to patient selection. Current evidence supports the feasibility of minimally invasive pCCA surgery in carefully selected patients treated at experienced centers but does not establish LS or RAS as superior alternatives to OS.

Furthermore, standardized indications for minimally invasive pCCA surgery remain undefined. Future prospective multicenter studies should incorporate detailed reporting of tumor characteristics, Bismuth classification, vascular involvement, extent of hepatectomy, caudate lobectomy, reconstruction requirements, institutional volume, surgeon experience, and adjusted long-term oncological outcomes to clarify the appropriate role of minimally invasive approaches.

## Data Availability

The original contributions presented in the study are included in the article/Supplementary Material, further inquiries can be directed to the corresponding author.
